# Post-inflammatory Isolated Bilateral Palatal Palsy Following Enteric Fever: A Report of a Rare Pediatric Case

**DOI:** 10.7759/cureus.111734

**Published:** 2026-06-29

**Authors:** Aboli Dahat, Girish Nanoti, Shraddha Tripathi

**Affiliations:** 1 Department of Pediatrics, NKP Salve Institute of Medical Sciences and Lata Mangeshkar Hospital, Nagpur, IND

**Keywords:** enteric fever, isolated bilateral palatal palsy, nasal regurgitation, nasal speech, post-inflammatory

## Abstract

Isolated acquired palatal palsy is an uncommon neurological condition in children and adolescents caused by selective dysfunction of the pharyngeal branch of the vagus nerve. Patients typically present with nasal regurgitation of liquids and feeds, hypernasal speech (nasal twang), and dysphagia due to impaired palatal movement. The condition is often considered post-infectious and has been associated with several viral and bacterial infections.

We report a rare case of isolated bilateral palatal paralysis following an enteric fever in a 17-year-old girl. She presented with the sudden onset of painless nasal regurgitation of liquids and feeds, accompanied by a nasal quality of speech. A comprehensive clinical evaluation was undertaken to exclude structural, infectious, and neuromuscular causes of palatal dysfunction. Magnetic resonance imaging of the brain revealed no abnormalities.

Isolated palatal palsy is an uncommon neurological disorder, and several viral and bacterial infections, including diphtheria, influenza, varicella, and enteric fever, have been implicated as potential precipitating factors. In the present case, the temporal association with enteric fever and the exclusion of other etiologies suggest a post-infectious immune-mediated neuropathy as the likely mechanism.

Based on the clinical presentation and the exclusion of structural, infectious, and neuromuscular causes, a diagnosis of post-infectious isolated bilateral palatal palsy was established. The child was commenced on oral methylprednisolone therapy. Significant clinical improvement was observed within three days of treatment initiation, with progressive resolution of symptoms thereafter. Complete recovery was achieved within 10 days, marked by the cessation of nasal regurgitation and restoration of normal speech. The rapid and favorable response to corticosteroid therapy further supported the presumed inflammatory or immune-mediated etiology of the condition.

Palatal palsy is a recognized neurological complication of enteric fever; however, most reported cases in the literature describe unilateral involvement. Bilateral palatal palsy is exceptionally rare, with only a few cases documented to date. The rarity of bilateral involvement makes the present case a unique clinical observation. Despite its rarity, early recognition is essential to avoid unnecessary investigations and to ensure timely treatment and recovery.

## Introduction

Isolated acquired palatal palsy is a rare neurological disorder in children and adolescents resulting from selective involvement of the pharyngeal branch of the vagus nerve. Clinically, it commonly presents with nasal regurgitation of feeds, hypernasal speech (nasal twang), and dysphagia. Although its exact etiology remains unclear, the condition is most frequently attributed to a post-infectious immune-mediated neuritis. Isolated palatal palsy generally follows a benign and self-limiting course, with most patients achieving complete recovery, either spontaneously or following corticosteroid therapy [1].

Our patient had a documented episode of enteric fever three weeks prior to presentation and remained asymptomatic after recovery until the onset of nasal regurgitation and hypernasal speech, suggesting a temporal association between the antecedent infection and the neurological manifestations. In this case, the acute onset of palatal paralysis following a recent febrile illness supports a post-infectious inflammatory neuropathy involving the vagus nerve (cranial nerve X). Several viral and bacterial infections, including diphtheria, influenza, varicella, and enteric fever, have been implicated as potential triggers of this immune-mediated process [2].

Owing to its rarity, isolated palatal palsy poses a diagnostic challenge and must be differentiated from more serious neurological conditions, including demyelinating neuropathies, brainstem lesions, and neuromuscular junction disorders [3]. Early recognition and appropriate management are essential to ensure a favorable outcome and prevent unnecessary investigations. Although several cases of isolated unilateral palatal palsy have been reported in the literature, bilateral palatal palsy remains exceedingly uncommon. Palatal palsy is a recognized neurological complication of enteric fever; however, the bilateral involvement observed in our patient distinguishes this case and enhances its clinical significance [4].

We report a case of a 17-year-old girl who developed isolated bilateral palatal palsy following enteric fever and demonstrated complete recovery with corticosteroid therapy.

## Case presentation

A 17-year-old girl presented to the outpatient department with complaints of nasal regurgitation of feeds for the preceding four to five days. The symptoms had a sudden onset and were painless in nature. The nasal regurgitation was associated with hypernasal speech (nasal twang) (Video 1). She also reported generalized weakness for the preceding three days.

**Video 1:** Video depicting a child speaking with a nasal twang of voice on presentation.

Prior to the onset of neurological symptoms, the child had a history of enteric fever. Approximately three weeks before presentation, she developed a high-grade fever associated with chills, rigors, and headache, without any associated rash. Investigations revealed a positive Widal test (H titer: 1:160, O titer: 1:160), and blood culture subsequently grew *Salmonella Typhi*, confirming the diagnosis of enteric fever. The child was treated with IV antibiotics (ceftriaxone) for 10 days, resulting in complete resolution of fever.

There was no preceding history of sore throat, rhinorrhea, cough, nasal obstruction, mouth breathing, or epistaxis. The child did not report any facial weakness, tongue deviation, limb weakness, or respiratory difficulty.

The child was immunized appropriately for age. There was no history of recent vaccination or travelling, preceding trauma, persistent headache, vomiting, seizures, altered sensorium, diplopia, sensory disturbances, or bowel or bladder dysfunction. Her past medical history was unremarkable, and there was no significant family history of neurological, systemic, or autoimmune disorders.

On examination, the child was conscious, alert, and oriented, with stable vital parameters. Neurological examination revealed hypernasal speech (nasal twang) and nasal regurgitation of feeds. Oropharyngeal examination demonstrated bilateral symmetrical palatal weakness with absent elevation of both sides of the soft palate during phonation (absent palatal movements), the absence of uvular deviation (a centrally placed uvula), and an absent gag reflex, findings that are characteristic of bilateral rather than unilateral palatal involvement. There was no evidence of involvement of any other cranial nerves. Motor and sensory examinations of all four limbs were normal, and no cerebellar or meningeal irritation signs were present.

A comprehensive diagnostic evaluation was undertaken to exclude structural, infectious, and neuromuscular causes of palatal dysfunction. Magnetic resonance imaging of the brain was normal, with no evidence of brainstem, posterior fossa, or other intracranial abnormalities. Electrophysiological studies revealed no evidence of generalized neuropathy or neuromuscular junction disorder. Laboratory investigations, including infectious and autoimmune workup, were unremarkable and did not suggest any active infection or underlying autoimmune pathology.

Baseline laboratory investigations showed a hemoglobin level of 119 g/L, total leukocyte count of 10.5 × 10⁹/L, and CRP of 4 mg/L. Serological testing for neurotropic viral infections, including herpes simplex virus (HSV), cytomegalovirus (CMV), Epstein-Barr virus (EBV), varicella-zoster virus, human Immunodeficiency virus (HIV), and hepatitis A virus, was negative. Autoimmune workup, including rheumatoid factor (RF) and antinuclear antibodies (ANA), was unremarkable. CSF examination demonstrated normal cell counts and protein concentration, with no evidence of albuminocytological dissociation. Chest radiography and electrocardiogram (EKG) demonstrated no abnormalities.

Following a thorough clinical evaluation and exclusion of alternative etiologies, a diagnosis of post-infectious isolated bilateral palatal palsy was made. Oral methylprednisolone was commenced at a dose of 1 mg/kg/day. The patient demonstrated significant clinical improvement within three days of treatment initiation, with complete resolution of symptoms within 10 days. Recovery was marked by the cessation of nasal regurgitation, restoration of normal speech (Video 2), and reappearance of the gag reflex. No treatment-related adverse events were noted.

**Video 2:** Video depicting normal tone of voice after treatment with steroids.

## Discussion

Isolated acquired pharyngeal hemiparalysis has been documented earlier, affecting primarily males in their first or second decade of life. Though in our case, it was a bilateral paralysis in a female child, which is quite a rare occurrence. Usually, it presents with a nasal voice and nasal escape of fluids on the same side. The most common presenting features were hypernasal speech (97%), nasal reflux (73%), and dysphagia (49%) [5,6].

The increased susceptibility of the pediatric population to isolated cranial neuropathies has been attributed to the relative immaturity of neural tissues, rendering them more vulnerable to infectious and ischemic insults. The underlying pathophysiology is believed to involve transient demyelination of the pharyngeal branch of the vagus nerve or a post-infectious neuritic process [1]. Isolated palatal palsy is an uncommon cranial mononeuropathy that is most often associated with immune-mediated inflammation occurring after a preceding infectious illness [5]. Several viral pathogens have been implicated in its etiology, including EBV, Coxsackie virus, measles virus, varicella-zoster virus, parvovirus B19, and hepatitis A virus (HAV). In many cases, however, no definite causative agent can be identified, supporting the hypothesis of an immune-mediated post-infectious mechanism [7].

Enteric fever is associated with a wide spectrum of neurological complications; however, isolated palatal or pharyngeal paralysis remains an exceptionally rare manifestation. The neurotoxicity of *Salmonella Typhi* has been attributed to the lipid A component of its lipopolysaccharide endotoxin, which may contribute to neurological involvement. Although any part of the central nervous system can be affected in enteric fever, cranial neuropathies such as palatal palsy are infrequently reported [3].

In a large series of 316 African and Indian children with enteric fever, Scragg et al. did not identify a single case of palatal paralysis, highlighting the rarity of this complication [8]. Furthermore, neurological manifestations of enteric fever generally occur early in the course of the illness [9]. In contrast, our patient developed bilateral pharyngeal paralysis on the 15th day of illness, suggesting a delayed neurological complication. This differs from the case reported by Jaiswal et al., in which palatal paralysis developed on the fifth day of illness [10]. The delayed onset in our patient may support a post-infectious immune-mediated mechanism rather than direct neurotoxicity.

The differential diagnoses of isolated pharyngeal paralysis include brainstem stroke or neoplasm, Guillain-Barré syndrome, myasthenia gravis, and diphtheritic neuropathy [5]. In the present case, these conditions were excluded on the basis of the clinical presentation and appropriate laboratory and radiological investigations. The rapid and complete recovery following corticosteroid therapy further supports an underlying inflammatory or immune-mediated etiology [2].

Given the excellent prognosis of this condition, with a high rate of complete recovery and a low risk of recurrence, invasive diagnostic procedures should be avoided whenever possible [11]. Most reported cases demonstrate a favorable response to corticosteroid therapy, although spontaneous recovery has also been documented in the absence of treatment [12]. Previous studies have shown that clinical improvement typically begins within 10 days of symptom onset, with complete resolution of pharyngeal weakness occurring within four to seven weeks, irrespective of steroid administration [13]. Consistent with these observations, our patient showed marked clinical improvement following corticosteroid therapy and achieved complete recovery on follow-up.

Most reported cases of isolated palatal palsy in children follow a benign clinical course, with either spontaneous recovery or a favorable response to corticosteroid therapy. Early recognition of this rare neurological entity is essential to avoid unnecessary investigations and alleviate parental anxiety. Prompt initiation of immunomodulatory treatment, when indicated, may hasten recovery and contribute to excellent functional outcomes [1,5]. The present case highlights the importance of considering post-infectious cranial neuropathy in children presenting with acute-onset palatal paralysis following enteric fever and reinforces the generally favorable prognosis associated with this condition.

## Conclusions

Isolated bilateral palatal paralysis is an exceedingly rare neurological condition that underscores the importance of a thorough cranial nerve examination and detailed assessment of the oral peripheral mechanism in children presenting with dysphagia, nasal regurgitation, or hypernasal speech. The present case highlights the need to consider post-infectious immune-mediated neuropathy as a potential mechanism underlying bilateral palatal palsy.

As demonstrated in this case and supported by previous reports, early recognition and appropriate corticosteroid therapy may facilitate rapid recovery and complete remission, although spontaneous resolution has also been documented owing to the self-limiting nature of the condition. Given the temporal association between culture-confirmed enteric fever and the onset of neurological symptoms, *Salmonella Typhi* infection should be considered in the differential diagnosis of isolated palatal or pharyngeal paralysis, particularly in regions where enteric fever remains endemic. Further studies are required to better elucidate the pathophysiological mechanisms, establish the role of immunomodulatory therapy, and develop evidence-based management strategies for this rare clinical entity.
